# Editorial: Old habits die hard: from risk calculators and stenosis evaluation to phenotyping coronary atherosclerosis using cardiac CT

**DOI:** 10.3389/fcvm.2023.1235144

**Published:** 2023-06-22

**Authors:** Leandro Slipczuk, Matthew J. Budoff, Todd C. Villines

**Affiliations:** ^1^ Division of Cardiology, Montefiore Medical Center/Albert Einstein College of Medicine, Bronx, NY, United States; ^2^ Department of Medicine, Lundquist Institute at Harbor UCLA Medical Center, Torrance, CA, United States; ^3^ Cardiovascular Medicine, University of Virginia, Charlottesville, VA, United States

**Keywords:** cardiac CT, coronary plaque, coronary stenosis, plaque burden and plaque calcification, coronary calcium score

**Editorial on the Research Topic** Beyond coronary stenosis: from diagnosis to therapy

Clinical atherosclerotic cardiovascular disease (ASCVD) is associated with high morbidity and mortality (1) with a substantial economic burden on the US health system, which is expected to reach $509 billion by 2035 (2). Forecasting the development of ASCVD has proved to be challenging because of the interaction and variation of multiple risk factors over time. Ranging from the primordial to secondary, ASCVD prevention represents one of the cornerstones of modern cardiology (3). Although statins are inexpensive and associated with a low incidence of side effects, the problem with a treat-all strategy is 2-fold. First, many physicians and asymptomatic individuals are unwilling to commit to a life-long medication because of the suboptimal therapeutic yield; second, risk heterogeneity necessitates the identification of higher-risk individuals to maximize the net benefit of additional expensive medications.

The implementation of prediction scores to identify high-risk individuals has been endorsed by multiple guidelines to initiate or intensify preventative treatments (4, 5). Purely clinical scores have several limitations, such as over- or underperformance in racial/ethnic minorities, the lack of accounting for dynamic changes occurring in risk factors, and the over-reliance on age (6). These limitations result in overconfidence without an acknowledgment of the real short- and long-term cardiovascular risks, potentially leading to over/undertreatment with preventative treatments. Moreover, the identification of obstructive coronary stenosis has traditionally served as the primary focus of risk stratification in patients undergoing functional stress testing and invasive coronary angiography. While there are many types of investigation aimed to improve the technique of cardiovascular prevention in individuals, such as the use of polygenic risk scores and biomarkers, there is clear evidence in the literature that it is the amount and type of coronary artery plaque that provides the most accurate and personalized assessment of an individual's risk for myocardial infarction (MI) (7).

It is now accepted that coronary artery calcium scoring (CAC) can provide an estimation of plaque burden and reclassify a large proportion of asymptomatic individuals into more accurate risk categories when compared with probabilistic clinical risk calculators (8). Coronary artery calcium scoring is particularly useful in borderline and intermediate risk groups, with superior derisking power (with CAC = 0), when compared with other proposed biomarker tests (9). Moreover, it can identify individuals without prior ASCVD at an equivalent risk of major cardiovascular events to those with established ASCVD (10). Nevertheless, the presence of risk factors remains an essential element when assessing the implications of CAC, particularly in young individuals (11). In patients with chest pain, CAC can act as a gatekeeper to CT coronary angiography (CCTA) or further diagnostic testing modalities with a high negative predictive value to exclude obstructive CAD and clinical events (12). However, a percentage of patients with CAC = 0 may have a non-calcified plaque [up to 16% in the PROMISE trial (13)], representing a missed opportunity for preventative therapies.

In recent large-randomized clinical trials, such as the PROMISE and SCOT-HEART, the use of CCTA in symptomatic patients was associated with a lower risk for myocardial infarction than conventional management, mostly due to the intensification of preventative therapies (14, 15). Importantly, in the SCOT-HEART trial, these findings were independent of CAC and were noted in patients with non-cardiac chest pain, suggesting a value in plaque detection to guide the initiation of preventative therapies in asymptomatic individuals. We now have three reasonably large-scale population-based studies on CCTA imaging in asymptomatic individuals [SCAPIS (*N* = 25,182) (16), Miami Heart (*N* = 2,459) (17), and Copenhagen General Population Study (*N* = 9,533) (18)], each demonstrating an overall high burden of subclinical coronary artery disease (>42%) in presumed low-risk asymptomatic populations, most of whom would not have otherwise qualified for preventative treatment such as statins.

The Copenhagen heart study demonstrated in more than 9,500 healthy subjects (57% women) that 46% had CAD to some extent, 10% had a stenosis ≥50% (“obstructive disease”), 10% had “extensive” disease (defined as plaque present in one-third of or more coronary segments), and 5% had both obstructive and extensive CAD. The subjects were followed up for up to a median of 3.5 years for determining the primary outcome of incident MI and the secondary composite outcome of MI or death. As expected, both stenosis and extensiveness of CAD significantly impacted event-free survival rates. The risk of death or myocardial infarction was increased in persons with extensive disease, regardless of the degree of obstruction—non-obstructive-extensive [adjusted relative risk, 2.70 (CI, 1.72−4.25)] and obstructive-extensive [adjusted relative risk, 3.15 (CI, 2.05−4.83)]. Importantly, subjects and providers were blinded to the results of CCTA, and only 17% with coronary atherosclerosis were on statins during the course of the study, highlighting the risk of minimally treated, unrecognized coronary atherosclerosis even in patients presumed to be at lower risk.

**Figure 1:**
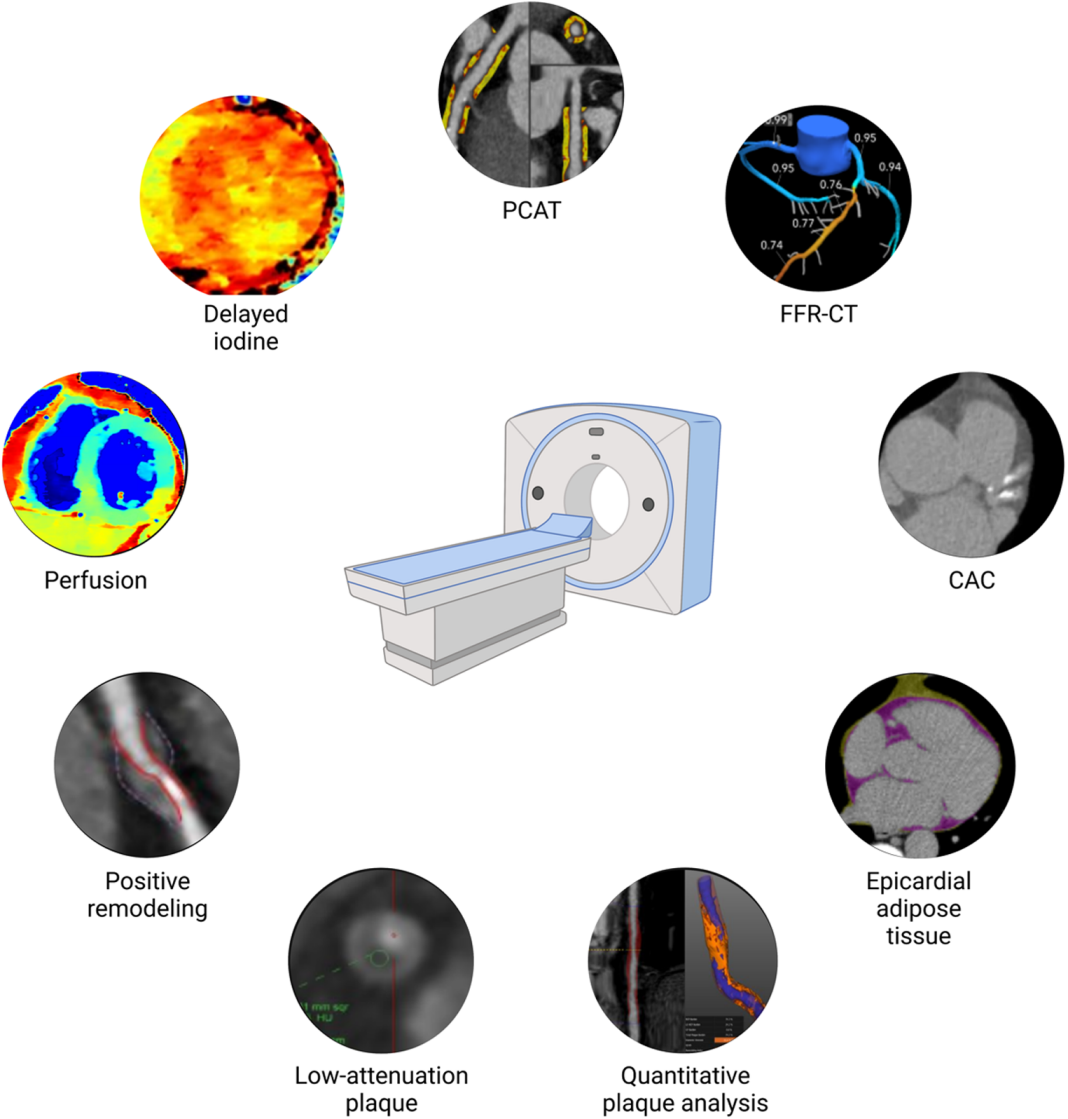
Coronary atherosclerosis phenotype by cardiac computed tomography. CAC, coronary artery calcium scoring; FFR-CT, fractional flow reserve calculation derived from computed tomography angiography; PCAT, pericoronary adipose tissue.

Beyond stenosis, assisted by novel artificial intelligence–driven platforms, CCTA can evaluate plaque burden (19) and characteristics (20, 21) with high efficiency and accuracy quantitatively (Figure 1). In addition, it can evaluate epicardial (EAT) (22) and pericoronary adipose tissue (PCAT) (23), which are inflammation imaging markers found to be associated with coronary atherosclerosis and myocardial infarction. These types of phenotypic characterization with CCTA raises the question whether they could potentially be utilized for tailoring preventative therapies to a specific risk phenotype.

Articles published in this research topic align with this quest. Ota et al. found in 107 patients who underwent CCTA and percutaneous coronary intervention (PCI) that lipid-rich plaque by near-infrared spectroscopy intravascular ultrasound and CCTA could predict myocardial injury during PCI (CT density was, however, not found to be an independent predictor). Zhao et al. demonstrated in 523 patients with diabetes mellitus and chronic coronary syndrome who underwent CCTA and PCI, that half of the major atherosclerotic cardiovascular events (MACE) were attributable to non-culprit lesions with high-risk plaque features as defined by CCTA. Jin et al. retrospectively studied 277 Chinese patients who underwent CCTA and invasive coronary angiography and found that EAT volume correlated with the presence and severity of hemodynamically significant CAD. Steyer et al. investigated the prognostic value of PCAT in patients undergoing cardiac CT for planning TAVR in 62 White patients from Germany and found that RCA PCAT attenuation prevailed as the only marker with a significant association with MACE.

In summary, the novel comprehensive phenotypic characterization by cardiac CT has promising clinical implications beyond stenosis assessment. Future studies are needed to assess the impact of phenotype-guided prevention on outcomes in patients from diverse sex/race/ethnicity backgrounds under different clinical scenarios.
